# Longitudinal associations of circadian eating patterns with sleep quality, fatigue and inflammation in colorectal cancer survivors up to 24 months post-treatment

**DOI:** 10.1017/S0007114523002714

**Published:** 2024-04-14

**Authors:** Marvin Y. Chong, Simone J. P. M. Eussen, Eline H. van Roekel, Gerda K. Pot, Annemarie Koster, Stéphanie O. Breukink, Maryska L. G. Janssen-Heijnen, Eric T. P. Keulen, Coen D. A. Stehouwer, Matty P. Weijenberg, Martijn J. L. Bours

**Affiliations:** 1 Department of Epidemiology, GROW School for Oncology and Reproduction, Maastricht University, Maastricht, The Netherlands; 2 Department of Epidemiology, CARIM School for Cardiovascular Diseases, Maastricht University, Maastricht, The Netherlands; 3 Department of Epidemiology, CAPHRI School for Care and Public Health Research, Maastricht University, Maastricht, The Netherlands; 4 Nutrition and Healthcare Alliance Hospital Gelderse Vallei, Ede, The Netherlands; 5 Department of Social Medicine, CAPHRI Care and Public Health Research Institute, Maastricht University, Maastricht, The Netherlands; 6 Department of Surgery, GROW School for Oncology and Reproduction, NUTRIM School of Nutrition and Translational Research in Metabolism, Maastricht University Medical Centre+, Maastricht, The Netherlands; 7 Department of Clinical Epidemiology, VieCuri Medical Centre, Venlo, The Netherlands; 8 Department of Internal Medicine and Gastroenterology, Zuyderland Medical Centre Sittard-Geleen, Geleen, The Netherlands; 9 Department of Internal Medicine, CARIM School for Cardiovascular Diseases, Maastricht University, Maastricht University Medical Centre, Maastricht, The Netherlands

**Keywords:** Colorectal cancer survivors, Chrono-nutrition, Diet, Fatigue, Insomnia, Sleep, Inflammation

## Abstract

Fatigue and insomnia, potentially induced by inflammation, are distressing symptoms experienced by colorectal cancer (CRC) survivors. Emerging evidence suggests that besides the nutritional quality and quantity, also the timing, frequency and regularity of dietary intake (chrono-nutrition) could be important for alleviating these symptoms. We investigated longitudinal associations of circadian eating patterns with sleep quality, fatigue and inflammation in CRC survivors. In a prospective cohort of 459 stage I-III CRC survivors, four repeated measurements were performed between 6 weeks and 24 months post-treatment. Chrono-nutrition variables included meal energy contribution, frequency (a maximum of six meals could be reported each day), irregularity and time window (TW) of energetic intake, operationalised based on 7-d dietary records. Outcomes included sleep quality, fatigue and plasma concentrations of inflammatory markers. Longitudinal associations of chrono-nutrition variables with outcomes from 6 weeks until 24 months post-treatment were analysed by confounder-adjusted linear mixed models, including hybrid models to disentangle intra-individual changes from inter-individual differences over time. An hour longer TW of energetic intake between individuals was associated with less fatigue (*β*: −6·1; 95 % CI (−8·8, −3·3)) and insomnia (*β*: −4·8; 95 % CI (−7·4, −2·1)). A higher meal frequency of on average 0·6 meals/d between individuals was associated with less fatigue (*β*: −3·7; 95 % CI (−6·6, −0·8)). An hour increase in TW of energetic intake within individuals was associated with less insomnia (*β*: −3·0; 95 % CI (−5·2, −0·8)) and inflammation (*β*: −0·1; 95 % CI (−0·1, 0·0)). Our results suggest that longer TWs of energetic intake and higher meal frequencies may be associated with less fatigue, insomnia and inflammation among CRC survivors. Future studies with larger contrasts in chrono-nutrition variables are needed to confirm these findings.

Colorectal cancer (CRC) is one of the leading causes of cancer-related morbidity and mortality worldwide^(1)^. As a consequence of the ageing population alongside advances in early detection and improved treatments, there are increasing numbers of CRC survivors, one of the most prevalent adult survivor populations^(2,3)^. Patients diagnosed with and treated for early stage I-III disease have 5-year survival rates of approximately 70–90 %^(2,3)^. Throughout this survivorship period, patients of all stages are faced with cancer-related and treatment-related side effects that negatively affect their overall quality of life^(4)^. Among the most common and distressing symptoms experienced by cancer patients are fatigue and sleep problems (insomnia), with up to two-thirds of CRC survivors affected^(5,6)^. Given the growing population of CRC survivors, identifying ways to decrease fatigue and to improve sleep quality is essential in the survivorship period.

Unhealthy lifestyles such as unhealthy diets are contributing to the rising incidence of CRC^(7)^. Diets rich in red and processed meats and sugar-sweetened beverages, but low in fruits, vegetables and fiber have been associated with an increased risk of CRC^(8)^. Moreover, these dietary factors have also been identified as factors that could negatively influence prognosis and quality of life after diagnosis^(8–10)^.

Previous research has shown that it is not only important what and how much we eat but also when, how often and how regular we consume food across the day^(11–14)^. Underlying the importance of the timing of nutritional intake are the human internal biological clocks. Many physiological and metabolic processes such as sleep/wake and immune functions show cyclic patterns across a period of approximately 24 h, which are referred to as circadian rhythms^(13,14)^. The internal timing systems responsible for the circadian rhythms integrate diverse environmental and metabolic stimuli, called ‘zeitgebers’, to regulate these processes^(15,16)^. For the central circadian clock, located in the superchiasmatic nucleus in the brain, light is the primary source for entrainment. From the superchiasmatic nucleus clock, information is transmitted to peripheral organ clock systems, mediating the synchronisation of internal body rhythms with external day and night cycles^(16)^. In addition, it has been demonstrated that the peripheral clock systems present in nearly all organs and tissues can be influenced by the consumption of food^(14)^. Consequently, a non-optimally timed diet could result in a misalignment between the peripheral and central clock systems. Acute or chronic periods of circadian misalignment could negatively influence sleep quality, sleep duration and fatigue and may even result in long-term negative health outcomes such as metabolic syndrome, in part as a result of increased inflammation^(17–21)^. Conversely, a well-timed dietary intake could potentially resolve a disrupted misalignment in the circadian system, by synchronising the peripheral clocks to the day and night cycle of light, thereby reducing the risk of negative health outcomes^(22,23)^.

The field of research that focuses on the interactions between the timing of food intake, the biological clock and health outcomes is called chrono-nutrition^(24,25)^. Chrono-nutrition comprises three aspects related to time: regularity, frequency and clock time of food intake, which all interact with circadian rhythms^(26)^. Concerning these aspects, certain factors have been associated with health benefits, potentially by positively influencing circadian rhythms: a regular meal pattern, consuming a higher proportion of energy in the beginning of the day, a meal frequency of 3 meals/d and regular fasting periods^(27,28)^. In addition, adhering to these behaviours could result in reduced inflammation, higher stress resistance and a lower risk for cardiometabolic disease^(12,27)^. Given the fact that insomnia, fatigue and the potentially underlying inflammation are all related to misalignments in circadian rhythms, and that the regularity, frequency and clock time of food intake interact with and could influence these rhythms, research in this area could have a high potential for symptom improvement. However, research into chrono-nutrition or circadian eating patterns is still in its infancy and especially lacking in CRC patients. Therefore, the aim of this explorative study was to investigate longitudinal associations of previously reported and newly operationalised circadian eating patterns with sleep quality, fatigue and inflammatory markers in CRC survivors from 6 weeks up to 24 months after the end of the cancer treatment.

## Methods

### Study design and population

**Fig. 1.**
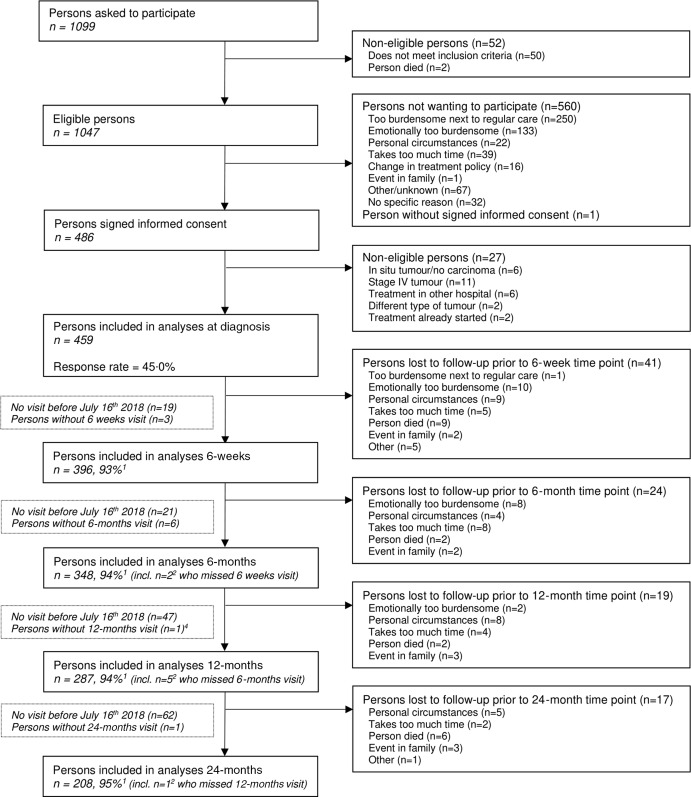
Flow diagram of inclusion of individuals within the Energy for Life after ColoRectal cancer (EnCoRe) study and included in the analyses of this article. Data of home visits performed before 16 July 2018 were included in the analyses. ^1^Response rate post-treatment = (persons included)/(persons included + persons lost to follow-up – persons died). ^2^Of the three persons without 6-week follow-up visits, one person did not have a 6-months follow-up visit before 16 July 2018. Of the six persons without 6-months follow-up visits, one person did not have a 12-months follow-up visit before 16 July 2018. This figure is published previously by Kenkhuis *et al*.^(10)^

Data were collected as part of the Energy for Life after ColoRectal cancer (EnCoRe) study, which is an ongoing prospective cohort study of CRC survivors in the Netherlands (Netherlands Trial Register number: NL6904)^(29)^. All patients diagnosed with stage I–III CRC at the Maastricht University Medical Center+ (Maastricht) from April 2012 onwards, and at VieCuri Medical Center (Venlo) and Zuyderland Medical Center (Sittard-Geleen) from 2014 onwards were eligible for inclusion. Patients, who had stage IV CRC, were younger than 18 years, were unable to read and speak the Dutch language, were not residing inside the Netherlands and who had comorbidities that could obstruct successful participation (e.g., Alzheimer’s disease) were excluded. This study was conducted according to the guidelines laid down in the Declaration of Helsinki, and all procedures involving human subjects/patients were approved by the Medical Ethics Committee of the University Hospital Maastricht and Maastricht University. All patients provided written informed consent prior to participation. A flow diagram describing the recruitment and follow-up of participants within the EnCoRe study, which were included in the analyses presented in the current paper, can be found in Fig. 1.

Participants were visited at their homes by trained dietitians who collected data after diagnosis before the start of treatment and at 6 weeks, 6 months, 12 months and 24 months post-treatment. In this regard, the start of treatment was either surgery or neo-adjuvant therapy, whereas post-treatment measurements referred to time after finishing initial therapy (surgery or adjuvant therapy). For the current analysis, data collected up until July 2018 were used. In total, information was available on 459 participants (response rate at baseline: 45 %) with follow-up measurements at 6 weeks (*n* 397), 6 months (*n* 348), 12 months (*n* 287) and 24 months (*n* 208) post-treatment. Response rates for the follow-up visits were all above 90 %, with the decreasing absolute numbers largely due to the fact that participants had not yet reached all post-treatment follow-up time points in July 2018^(10)^.

#### Dietary intake

Participants completed a 7-d structured dietary record at all post-treatment time points, but not at diagnosis, to provide quantitative data on food and beverage consumption at six predefined meal slots, i.e. three standard mealtimes (breakfast, lunch and dinner) and three snacking moments (morning snacking, afternoon snacking and evening snacking) in which one or more eating occasions, if any, in-between standard meals were accumulated. In the dietary record, consumed meals, foods and beverages were reported alongside details on brand names, portion sizes and preparation methods. In addition, eating times were available for the main meals of every reported day including clock times when participants had breakfast, lunch and dinner. No clock times were available for the snacking moments in-between main meals. Participants received detailed oral and written instructions on how to complete the dietary record. The dietitians involved checked the completed dietary records upon receipt, and contacted participants in those cases where information was incomplete or missing. Afterwards, daily dietary intake was calculated using food calculation software (Compl-eat; Wageningen University) based on the Dutch Food Composition database (NEVO-2011). Additional information regarding methods and procedures applied for the assessment and coding of dietary records are presented elsewhere^(30)^. Only participants with ≥four complete dietary record days including at least one weekend day were included in the analyses. Based on this criteria, two diaries (0·2 %) were excluded.

#### Fatigue

The validated twenty-item Checklist Individual Strength (CIS) was used at all post-treatment time points to obtain a comprehensive multidimensional assessment of fatigue^(31)^. The CIS is subdivided into four subscales: subjective fatigue (range: 8–56), motivation- (range: 4–28), concentration- (range: 5–35) and activity-related fatigue (range: 3–21)^(32)^. A total fatigue score was derived by summing all the subscales (possible range: 20–140). Higher scores for total fatigue and subscales of fatigue indicate worse fatigue. Fatigue was also assessed by use of the fatigue symptom scale from the European Organization for the Research and Treatment of Cancer Quality of Life Questionnaire (EORTC QLQ-C30)^(33)^. This cancer-specific and well-validated EORTC QLQ-C30 fatigue subscale contains three items, on the basis of which a fatigue score was calculated ranging from 0 to 100, with higher scores indicating more fatigue^(33)^.

#### Sleep quality

Participants’ sleep quality was assessed at all post-treatment time points using the validated single-item insomnia scale of the EORTC QLQ-C30^(33)^. The item included the question ‘Have you had trouble sleeping?’, which was answered with one of four alternatives from 1 ‘not at all’ to 4 ‘very much’. The scale was transformed to a range from 0 to 100, with higher values representing greater sleep problems (insomnia). A recent study has shown that the one-item sleep instrument of the EORTC QLQ-C30 is sufficient for group level investigations and reported a high correlation with an alternative questionnaire to detect sleep problems^(34)^. Sleep duration was measured by participants writing down their wake time and bedtime in the structured dietary record and was available for seven days. Additionally, in case of missing sleep times, the sleep duration was checked using the validated triaxial MOX activity meter (Maastricht Instruments B.V., Maastricht, the Netherlands), which participants wore on the anterior upper thigh 10 cm above the knee for seven consecutive days (24 h/d) at every post-treatment time point. Accelerometer data were deemed valid when there was ≥10 h of waking wear time/d, and only participants with ≥four valid days were included in the analyses.

#### Inflammatory markers

Blood samples were collected at diagnosis and at every post-treatment time point. All blood samples were centrifuged and aliquoted into plasma before being immediately stored in a freezer at –80°C until analysis. Plasma levels were quantified for the inflammatory markers interleukin 6 (IL6, pg/ml), IL8 (pg/ml), IL10 (pg/ml) and tumour necrosis factor *α* (TNF*α*, pg/ml) (Meso Scale Diagnostics, Rockville, MD, USA), and high-sensitivity C-reactive protein (hsCRP, µg/ml), neopterin (nmol/l), kynurenine (µmol/l) and tryptophan (µmol/l) (BEVITAL, Bergen, Norway). In addition, the kynurenine to tryptophan ratio was used as an inflammatory marker^(35)^. A summary inflammatory *Z*-score was then calculated so as to cluster conceptually related markers of low-grade inflammation and to improve the statistical efficiency^(36)^. This score included IL6, IL8, IL10, TNFα and hsCRP and was calculated as follows: for each individual, at each time point, a *Z*-score was calculated for all mentioned inflammatory markers (which were first log transformed) individually according to the formula: (individual value − population mean)/population standard deviation. As IL10 represents an anti-inflammatory cytokine, the individual *Z*-score for IL10 was multiplied by minus one to account for this anti-inflammatory effect. Afterwards, the resulting individual biomarker Z-scores were averaged into an overall sum score.

#### Lifestyle, clinical and socio-demographic factors

Socio-demographic characteristics of participants were retrieved from medical records, including age, sex and clinical information (i.e. cancer stage, cancer treatment received and tumor site). Self-reported data were collected on educational level (at diagnosis), current smoking status, presence of a stoma and on use of non-steroidal anti-inflammatory drugs (all time points). Additionally, the presence of comorbidities was assessed using the Self-Administered Comorbidity Questionnaire at all time points^(37)^. BMI (kg/m^2^) was determined based on body height (at diagnosis) and the average of duplicate body weight measurements, taken by trained dietitians at every time point. BMI was categorised according to the WHO guidelines into underweight (<18·5 kg/m^2^), normal weight (18·5–24·9 kg/m^2^), overweight (25·0–29·9 kg/m^2^) or obesity (≥ 30·0 kg/m^2^)^(38)^. The Short QUestionnaire to ASsess Health-Enhancing Physical Activity (SQUASH) was used to assess self-reported time spent in physical activity, including moderate to vigorous physical activity, at all time points^(39)^. The MOX activity meter was used to objectively measure daily sedentary time, as described previously by Roekel *et al*.^(40)^ Finally, a diet quality score was calculated using dietary records, based on the five nutrition recommendations of the World Cancer Research Fund (WCRF) and the American Institute for Cancer Research guidelines^(41,42)^.

### Operationalisation of chrono-nutrition variables

**Fig. 2.**
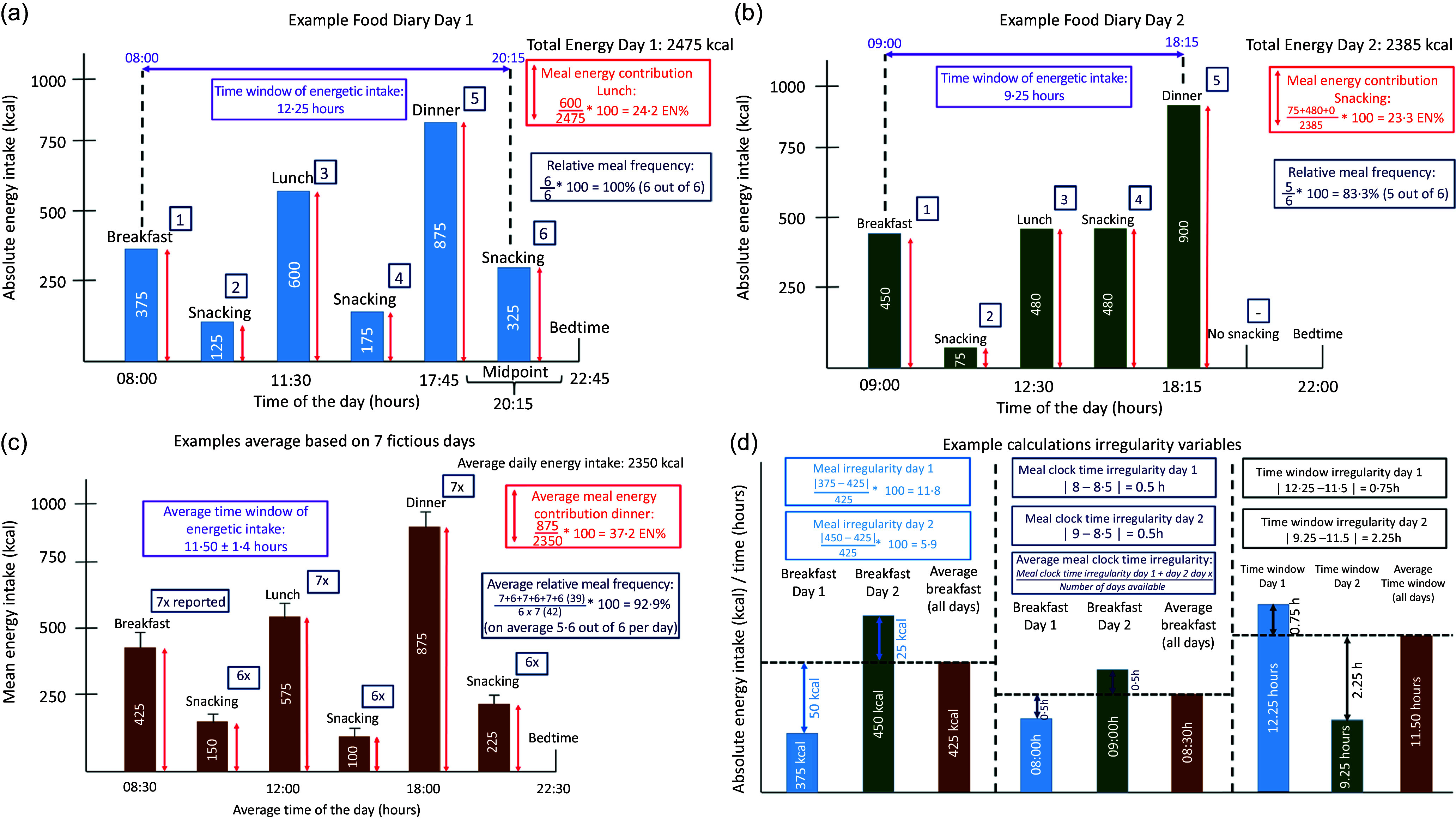
Visualisation of the operationalised chrono-nutrition variables in the current paper based on an example of 2 days of fictitious but realistic dietary record data. (a) Example of a first reported dietary record day for a fictional participant. For relative meal frequency, the number of meals slots in which energetic intake was reported was expressed as a percentage out of the maximum number of meal slots that could have been reported. The maximum is six meal slots/d, and because also six meal slots were reported, the relative meal frequency in this example is 100 %. The time window expresses the time in hours between the first and last occasion of energetic intake. In this study, clock times were only available for the main meals breakfast, lunch and dinner in the dietary record, and not for morning, afternoon and evening snacking. In case of reported snacking after dinner, the clock time of the last energetic intake was estimated as the midpoint between the clock time of dinner and the reported bedtime. In case of breakfast skipping, the clock time of the first energetic intake was the clock time of lunch when this was the first reported meal of the day. When snacking was reported before lunch, the clock time of the first energetic intake was estimated as the midpoint between the reported wake time and clock time of lunch. (b) Example of a second reported dietary day for a fictional participant. (c) Example of the average dietary record day based on all seven available reported dietary record days. The numbers mentioned here could be slightly different compared to what is expected based on the two example days shown, as this average was based on seven fictitious days. (d) Illustration of the chrono-nutrition variables meal irregularity, meal clock time irregularity and time window (TW) of energetic intake irregularity. Similar as shown for meal clock time irregularity, an average score was calculated for meal irregularity and TW irregularity. The irregularity values of individual dietary record days were summed and then divided by the total number of days available to obtain the average.

As described by Pot *et al*.^(26)^, chrono-nutrition includes multiple aspects related to time: regularity, frequency and clock time of food intake. In order to capture all these aspects and therefore circadian eating patterns in our population of CRC survivors, several chrono-nutrition variables were constructed. First, meal energy contribution variables were calculated for each of the specified meal slots, specifying the relative amount of energy (% of daily total) consumed at each meal slot during the day. Second, two meal irregularity scores were calculated, one based on the irregularity of energy intake during meal slots on repeated days^(26)^ and the other based on the irregularity of clock times the meals were consumed across the week. Third, a time window (TW) of energetic intake was calculated, illustrating the time between the first and last energetic intake of the day. Additionally, an irregularity score was calculated for the TWs of energetic intake across the week. Finally, the frequency of energetic intake (meal frequency) was calculated by counting the meal slots in which anything except for exclusively water was consumed. The chrono-nutrition variables are described in more detail below and in Fig. 2.

#### Meal energy contribution

Meal energy contribution was determined for every meal slot (breakfast, morning snacking, lunch, afternoon snacking, dinner and evening snacking) by dividing the energy intake (kcal)/d during a specific meal slot by the total daily energy intake, multiplying by 100 to obtain the percentage of daily energy consumed during that meal slot and averaging over all available days.

#### Meal irregularity score

Meal irregularity scores, describing the irregularity of meals based on variability in energy intake, were calculated for energy intake per meal slot and total daily energy intake. First, for calculating meal irregularity scores per meal slot, the absolute difference per available day was determined between energy intake during a specific meal slot and the mean weekly energy intake during that meal slot based on the number of available days. This absolute difference was divided by the mean weekly energy intake during that meal slot, multiplied by 100 and then averaged over all available days^(26)^. The meal energy irregularity score is a measure of day-to-day variation in energy intake per meal slot, with a higher score indicating a more irregular pattern of energy intake. Second, the meal irregularity score for total daily energy intake was calculated similarly, using total energy intake/d instead of energy intake per meal slot.

#### Meal clock time irregularity score

Meal clock time irregularity scores, describing the irregularity of meals based on variability in clock time of consumption, were calculated based on the clock times of breakfast, lunch and dinner meal slots. For each of these meal slots, the absolute difference per available day was determined between the clock time of a specific meal slot and the mean weekly clock time of that meal slot based on the number of available days. Afterwards, the absolute difference was averaged over the number of available days. The meal clock time irregularity score is a measure of day-to-day variation in the clock times the main meals are consumed, with higher scores indicating a more irregular meal intake pattern in terms of clock times. Only participants with ≥four individual day clock times of breakfast, lunch or dinner available were included in the analyses. If one of the meals was skipped and therefore no clock time was available, this day was not taken into account to calculate the irregularity score based on the variability in clock times.

#### Time window of energetic intake

The TW of energetic intake, defined as the time (in hours) between the first and last energetic intake of the day, was calculated and afterwards averaged over all available days. In case no clock times were available for the first or last energetic intake of the day (i.e. when morning or evening snacking was reported as first or last energetic intake, respectively), the average time between waking time and lunch time and the average time between dinner and bed time were used as clock time for the first and last energetic intake, respectively. The average was assumed to be the most accurate estimate of the true clock time in our population^(43)^. In this regard, anything except for consuming exclusively water was considered as energetic intake. Only participants with ≥four individual day TWs of energetic intake available were included in the analyses.

#### Irregularity of time window of energetic intake

The time-window irregularity score was calculated by first determining the absolute difference between the TW per available day and the mean weekly TW based on the number of available days. Afterwards, the absolute difference was averaged over the number of available days. The TW irregularity score presents a measure of day-to-day variation in the TW of energetic intake, with higher scores reflecting a more irregular TW of energetic intake. Only participants with ≥four individual day TWs of energetic intake available were included in the analyses.

#### Relative meal frequency

For calculating meal frequency, the meal slots in which energetic intake was reported were first summed for every participant across all available days of the dietary record. The maximum number of eating occasions was six for every available day, including the meal slots breakfast, morning snacking, lunch, afternoon snacking, dinner and evening snacking. Next, the total number of meal slots in which energetic intake was reported across all available days was divided by the maximum number of eating occasions, which was determined by multiplying the number of available days by six, the maximum number of eating occasions on one day. The outcome was then multiplied by 100 to obtain the relative meal frequency as a percentage of the maximum number of possible eating occasions.

### Statistical analysis

Descriptive analyses were performed to ascertain main baseline characteristics for the study population, including socio-demographic, lifestyle and clinical variables at every time point. Values are presented as mean (±sd) for normally distributed quantitative variables, as median (25th–75th percentile) for non-normally distributed quantitative variables, or as frequency (%) of categorical class.

Confounder-adjusted linear mixed models were used to analyse the longitudinal associations of each of the chrono-nutrition variables in relation to sleep quality, fatigue and inflammatory markers between 6 weeks and 24 months post-treatment. All chrono-nutrition variables were modeled continuously. Based on literature, associations were adjusted for an *a priori* defined set of potential confounders including age at enrollment (in years), sex, time since end of treatment (weeks), number of comorbidities (0, 1, ≥2), chemotherapy (yes/no), presence of a stoma (yes/no), diabetes type 1 or 2 (yes/no), BMI (kg/m^2^), total energy intake (kcal/week), diet quality (WCRF/AICR score) and moderate to vigorous physical activity (min/week). Additionally, the 10 % change-in-estimate method^(44)^ was used to explore the influence of an additional set of potential confounders including education (low, medium and high), radiotherapy (yes/no), partner status (yes/no), smoking (current, former, never), prolonged sedentary behaviour (h/d) and alcohol intake (g/d). Sedentary behaviour and alcohol intake led to a > 10 % change in most *β* estimates and were therefore included as confounders to calculate the fully adjusted association. The use of random slopes was tested with a likelihood ratio test, and random slopes were added when the model fit improved statistically significantly. Furthermore, hybrid modelling was used to disaggregate the inter- and intra-individual associations by adding both centered person-mean values and individual deviations from the person-mean values, respectively^(45)^. Potential interaction between chrono–nutrition variables and sex was explored by including interaction terms into the linear mixed models. No statistically significant interaction effects were found.

Time-lag analyses using linear mixed models were performed to explore the directionality of the associations in cases where exposures showed a clear pattern of significant associations with either one or more outcomes. This was done by modeling how the exposure measurements at 6-week, 6-month and 12-month time points were related to the outcome measurements at the 6-month, 12-month and 24-month time points, respectively, while including time-varying confounder information at the same times as the exposure measurements.

A sensitivity analysis was performed to determine the influence of small eating occasions (<50 kcal) on the investigated associations, as these could severely influence meal frequency and irregularity scores. Specifically, all eating occasions with <50 kcal were omitted, whereafter chrono-nutrition variables were recalculated, and the linear mixed models analyses with sleep quality, fatigue and inflammatory markers as outcomes were re-run. All statistical analyses were performed with the use of Stata 15.0 (StataCorp. LLC) with the statistical significance set at *P* < 0·05 (two-sided).

## Results

The participant characteristics of the total population at diagnosis (*n* 459) and at the follow-up time points are presented in Table 1. From this study population, 397 were included in the analyses (at 6 weeks to potentially 24 months post-treatment). Response rates were above 90 % at all follow-up time points; the decreasing absolute numbers were largely due to the fact that participants had not yet reached all post-treatment follow-up time points at the time of data analysis (Fig. 1). At diagnosis, participants were on average 66·9 (sd = 9·1) years old and 66 % of participants were male. Approximately half of the participants were diagnosed with stage III cancer (45·8 %), followed by stage I cancer (30·7 %) and stage II cancer (23·5 %). With regard to treatment, 25·3 % of participants received radiotherapy, of which the majority received pre-operative radiotherapy and only two participants received post-operative radiotherapy. In total, 18·3 % of participants received pre-operative chemotherapy and 28·5 % of participants received post-operative chemotherapy. In total, 40·1 % of participants received chemotherapy since some participants received both pre- as well as post-operative chemotherapy. Pre-operative chemotherapy consisted out of Capacetabine monotherapy, whereas post-operative chemotherapy consisted mostly (86·3 %) out of capacetabine + oxaliplatin (CAPOX). In addition, 9·2 % of participants received capacetabine monotherapy and one person received 5FU + oxaliplatin (FOLFOX) as post-operative chemotherapy.

**Table 1.** Socio-demographic, lifestyle and clinical characteristics of the study population of colorectal cancer survivors from diagnosis up to 24 months after treatment

Characteristics	Diagnosis (*n* 459)		6 weeks post-treatment (*n* 397)		6 months post-treatment (*n* 348)		12 months post-treatment (*n* 287)		24 months post-treatment (*n* 208)*	
	Mean	sd	Mean	sd	Mean	sd	Mean	sd	Mean	sd
Age, years	66·9	9·1	67·0	9·1	67·2	9·2	67·4	9·2	68·1	9·2
	*n*	%	*n*	%	*n*	%	*n*	%	*n*	%
Sex (male)	303	66·0	271	68·4	236	67·8	195	67·9	143	68·8
Education										
Low	130	29·0	107	27·0	91	26·2	73	25·5	45	21·6
Medium	168	37·4	150	37·9	137	39·5	114	39·9	89	42·8
High	151	33·6	139	35·1	119	34·3	99	34·6	74	34·6
	Mean	sd	Mean	sd	Mean	sd	Mean	sd	Mean	sd
BMI, kg/m^2^	28·3	4·7	27·8	4·6	28·3	4·7	28·7	4·8	28·3	4·6
Underweight: <18·5	2	0·4	2	0·5	0	0·0	1	0·4	1	0·5
Healthy weight: 18·5–24·9	111	24·3	117	29·6	90	25·9	62	21·9	49	24·0
Overweight: 25·0–29·9	201	44·0	173	43·8	151	43·5	130	45·9	85	41·7
Obese: ≥ 30	143	31·3	103	26·1	106	30·6	90	31·8	69	33·8
	*n*	%	*n*	%	*n*	%	*n*	%	*n*	%
Partner (yes)	360	80·2	309	79·6	274	80·6	224	80·3	161	81·3
Stoma (yes)	3	0·7	110	28·4	68	19·8	43	15·2	26	13·1
Treatment										
Surgery	412	89·8	355	89·4	314	90·2	259	90·2	186	89·4
Received chemotherapy	184	40·1	156	39·3	134	38·5	108	37·6	80	38·5
Received radiotherapy	116	25·3	102	25·7	88	25·3	74	25·8	56	26·9
Cancer type										
Colon	290	63·2	250	63·0	222	63·8	181	63·1	125	60·0
Rectosigmoid and rectum	169	36·8	147	37·0	126	36·2	106	36·9	83	40·0
Tumour stage										
Stage I	141	30·7	124	31·2	109	31·3	97	33·8	71	34·1
Stage II	108	23·5	100	25·2	86	24·7	68	23·7	51	24·5
Stage III	210	45·8	173	43·6	153	44·0	122	42·5	86	41·3
Comorbidities										
0 comorbidities	–		91	23·0	88	25·4	71	25·1	46	22·6
1 comorbidity	–		102	25·7	87	25·1	64	22·6	49	24·0
≥2 comorbidities	–		203	51·3	172	49·6	148	52·3	109	53·4
	Median	IQR	Median	IQR	Median	IQR	Median	IQR	Median	IQR
MVPA, min/week	660	780	420	645	570	660	600	750	600	765
	Mean	sd	Mean	sd	Mean	sd	Mean	sd	Mean	sd
Prolonged sedentary time, h/d	–		5·3	2·7	4·2	1·9	4·4	1·9	4·5	1·9
	*n*	%	*n*	%	*n*	%	*n*	%	*n*	%
Smoking status										
Never	139	31·0	118	30·5	98	28·7	76	27·6	57	29·1
Former	255	56·8	235	60·7	213	62·5	172	62·6	120	61·2
Current	55	12·3	34	8·8	30	8·8	27	9·8	19	9·7

MVPA, moderate-to-vigorous physical activity.

* Response rates for the follow-up time-points were all above 90 %. The decreasing absolute numbers are largely due to the fact that participants had not yet reached all post-treatment follow-up time points at the time of data analysis.

### Changes in chrono-nutrition variables and in sleep quality, fatigue and inflammatory markers from 6 weeks up to 24 months post-treatment

At 6 weeks after treatment, most daily energy was consumed during dinner (33·9 ± 7·8 EN %), followed by snacking (25·8 ± 9·7 EN %), lunch (22·4 ± 7·9 EN %) and breakfast (17·8 ± 6·3 EN %), respectively. The energy percentages consumed during the different meal moments remained relatively stable over time (Table 2). At 6 weeks after treatment, highest irregularity scores based on variability of energy intake during meals across days were observed for snacking (43·8 ± 24·0) and lunch (36·8 ± 26·8). At 6 weeks post-treatment, participants reported on average energetic intake in 5·5 out of 6 available meal slots/d. Both average irregularity scores based on variability of energy intake as well as meal frequency scores remained stable over time (Table 2). Participants’ TW of energetic intake followed a small but significant increase over time, ranging from 11·6 (±0·9) hours at 6 weeks to 11·8 (±1·1) hours at 24 months post-treatment (Table 2).

**Table 2.** Descriptive analyses of circadian eating patterns (meal energy contribution, meal irregularity, meal frequency and time window of energetic intake) and outcomes (sleep quality, fatigue and inflammatory markers) in the study population of colorectal cancer survivors from 6 weeks to 24 months post-treatment

Characteristics	6 weeks post-treatment		6 months post-treatment		12 months post-treatment		24 months post-treatment	
	Mean	sd	Mean	sd	Mean	sd	Mean	sd
Chrono-nutrition variables	*n* 383		*n* 332		*n* 275		*n* 195*	
Meal energy contribution								
Breakfast (EN %)	17·8	6·3	18·1	6·9	18·1	7·0	17·8	6·9
Lunch (EN %)	22·4	7·9	22·1	8·8	22·4	8·1	22·3	7·7
Dinner (EN %)	33·9	7·8	34·8	8·4	35·1	7·7	35·0	8·5
Snacking (EN %)	25·8	9·7	25·0	11·1	24·4	10·4	24·9	10·3
Meal irregularity – based on energy								
Breakfast	23·7	22·2	24·7	19·9	23·2	19·6	23·9	24·0
Lunch	36·8	26·8	38·1	30·5	37·1	31·5	37·5	31·3
Dinner	31·2	14·7	32·1	15·6	31·3	14·9	32·1	15·0
Snacking	43·8	24·0	48·0	25·9	47·8	25·1	46·7	25·2
Across days	15·0	6·2	16·3	8·0	15·9	7·1	16·1	8·5
Meal frequency								
Out of a maximum of six reported meals/d	5·5	0·6	5·5	0·6	5·5	0·6	5·5	0·5
Meal irregularity – based on clock time	*n* 355		*n* 304		*n* 255		*n* 183	
Breakfast (hours)	0·5	0·4	0·5	0·4	0·5	0·4	0·5	0·4
Lunch (hours)	0·4	0·3	0·4	0·3	0·4	0·3	0·4	0·3
Dinner (hours)	0·4	0·3	0·4	0·4	0·4	0·4	0·4	0·3
Time window (TW) of energetic intake	*n* 360		*n* 312		*n* 255		*n* 181	
TW (hours)	11·6	0·9	11·6	1·0	11·7	1·1	11·8	1·1
Irregularity (hours)	0·8	0·5	0·9	0·5	0·8	0·5	0·8	0·5
EORTC QLQ-C30 (range 0–100)†	*n* 389		*n* 344		*n* 283		*n* 199	
Fatigue	27·6	22·9	22·4	21·0	20·5	22·2	19·3	22·2
Insomnia	22·5	28·3	19·0	26·6	19·1	26·6	19·6	27·0
Checklist individual strength (CIS)	*n* 388		*n* 344		*n* 283		*n* 199	
Total fatigue (range 20–140)	62·3	26·2	57·3	26·1	53·9	25·9	53·9	26·5
Subjective fatigue (range 8–56)	26·5	13·2	24·0	12·3	22·3	12·3	22·3	12·6
Motivation-related fatigue (range 4–28)	12·3	6·0	11·4	5·8	10·8	6·0	10·8	5·8
Activity-related fatigue (range 3–21)	10·7	5·1	9·3	5·2	8·7	5·1	8·4	5·1
Concentration-related fatigue (range 5–35)	12·7	7·3	12·7	7·4	12·0	6·8	12·4	7·3
Sleep	*n* 326		*n* 290		*n* 234		*n* 175	
Sleep duration (h/night)	8·7	1·1	8·6	1·1	8·5	1·2	8·5	1·1
Inflammatory markers	*n* 253		*n* 209		*n* 169		*n* 74	
	Median	IQR	Median	IQR	Median	IQR	Median	IQR
IL6 (pg/ml)	1·5	0·8, 2·2	1·2	0·8, 2·0	0·9	0·6, 1·4	0·9	0·5, 1·5
IL8 (pg/ml)	5·6	4·4, 7·4	5·2	4·4, 7·0	3·9	3·1, 4·8	4·8	3·8, 6·2
IL10 (pg/ml)	0·4	0·3, 0·5	0·4	0·2, 0·5	0·2	0·2, 0·4	0·2	0·1, 0·3
TNF*α* (pg/ml)	2·9	2·4, 3·8	2·8	2·3, 3·6	2·0	1·6, 2·5	2·0	1·6, 2·9
hsCRP (µg/ml)	2·1	1·2, 5·2	1·8	0·7, 4·4	1·7	0·8, 5·2	Not measured	
Neopterin (nmol/l)	15·9	12·1, 22·8	15·3	11·4, 21·4	14·8	11·6, 20·4	Not measured	
Kynurenine (µmol/l)	1·9	1·7, 2·2	1·8	1·6, 2·1	1·8	1·6, 2·2	Not measured	
Tryptophan (µmol/l)	66·1	59·9, 72·4	67·4	59·0, 75·2	68·2	61·0, 75·7	Not measured	
	Mean	SD	Mean	SD	Mean	SD	Mean	SD
Kynurenine to tryptophan (KTR) ratio (x1000)	31·1	10·0	30·1	11·9	28·3	8·1	Not measured	
Summary inflammatory *Z*-score	0·1	0·5	0·1	0·5	−0·2	0·6	−0·1	0·6

EN %, energy percentage; EORTC QLQ-C30, European Organisation for the Research and Treatment of Cancer Quality of Life Questionnaire.

* Response rates for the follow-up time-points were all above 90 %. The decreasing absolute numbers are largely due to the fact that participants had not yet reached all post-treatment follow-up time points at the time of data analysis.

† Higher scores on the EORTC QLQ-C30 and Checklist Individual Strength (CIS) reflect more symptoms (i.e., worse fatigue or sleeping problems).

Highest mean scores for fatigue and insomnia were observed at 6 weeks post-treatment, followed by a decline towards 6 months post-treatment, after which scores remained relatively stable up to 24 months post-treatment (Table 2). For sleep duration and inflammatory markers IL6, IL10, TNF*α*, hsCRP, neopterin and kynurenine to tryptophan ratio (KTR), a decrease was observed between 6 weeks and 24 months post-treatment (Table 2).

### Longitudinal associations of chrono-nutrition variables with sleep quality, fatigue and inflammatory markers

#### Meal energy contribution

Confounder-adjusted longitudinal associations from 6 weeks to 24 months post-treatment show that a higher percentage of daily energy consumed during breakfast was statistically significantly associated with a longer sleep duration (*β* per 10 EN %: 0·2 h; 95 % CI (0·0, 0·3)) over time (Table 3). A higher percentage of daily energy consumed during dinner was significantly associated with a decrease in fatigue on the EORTC QLQ-C30 subscale (*β*: −1·9; 95 % CI (−3·4, −0·3)) and with total fatigue as measured by the CIS albeit non-significantly (*β*: −1·2; 95 % CI (−2·9, 0·5)). On the contrary, an increase in the percentage of daily energy consumed during snacking was significantly related to an increase in fatigue as measured by the EORTC QLQ-C30 subscale (*β*: 2·0; 95 % CI (0·6, 3·3)), and by the CIS albeit non-significantly (*β*: 1·3; 95 % CI (−0·2, 2·8)). A higher percentage of daily energy consumed during dinner was significantly associated with a lower inflammatory *Z*-score (*β*: −0·1; 95 % CI (−0·1, 0·0)). While a higher percentage of daily energy consumed during snacking was significantly associated with an increase in the inflammatory *Z*-score (*β*: 0·1; 95 % CI (0·0, 0·1)).

**Table 3.** Longitudinal associations of meal energy contribution with sleep quality, fatigue and inflammatory markers between 6 weeks and 24 months post-treatment

EN % intake during meal	CIS						EORTC QLQ-C30				Dietary record		Inflammatory markers	
For each 10 EN % increase	Total fatigue (20–140)		Subjective fatigue (8–56)		Activity fatigue (3–21)		Fatigue (0–100)		Insomnia (0–100)		Sleep duration		Summary *Z*-score	
	*β* *	95 % CI	*β*	95 % CI	*β*	95 % CI	*β*	95 % CI	*β*	95 % CI	*β*	95 % CI	*β*	95 % CI
Breakfast														
Adjusted†,‡	0·4	−2·0, 2·7	0·0	−1·1, 1·2	0·4	−0·1, 0·9	0·4	−1·6, 2·5	1·7	−1·1, 4·5	**0·2**	**0·0, 0·3**	0·0	−0·1, 0·1
Intra§	−1·0	−3·9, 1·9	−0·8	−2·2, 0·7	0·1	−0·5, 0·8	−0·1	−2·8, 2·6	1·6	−2·4, 5·5	**0·2**	**0·0, 0·3**	0·0	−0·1, 0·1
Inter‖	3·0	−1·0, 7·0	1·4	−0·5, 3·3	0·7	0·0, 1·5	1·2	−2·0, 4·3	1·8	−2·2, 5·7	0·1	0·0, 0·3	0·0	−0·1, 0·1
Lunch														
Adjusted†,‡	−0·8	−2·6, 1·1	−0·2	−1·2, 0·7	−0·1	−0·5, 0·3	−1·1	−2·7, 0·5	1·0	−1·2, 3·3	−0·1	−0·2, 0·1	0·0	−0·1, 0·0
Intra§	−0·2	−2·4, 2·1	0·0	−1·1, 1·2	0·3	−0·2, 0·8	−0·5	−2·6, 1·6	**3·7**	**0·7, 6·8**	0·0	−0·1, 0·1	0·0	−0·1, 0·1
Inter‖	−2·2	−5·5, 1·1	−0·8	−2·4, 0·7	**−0·8**	**−1·4, −0·1**	−2·1	−4·8, 0·5	−2·1	−5·4, 1·2	**−0·2**	**−0·3, 0·0**	0·0	−0·1, 0·0
Dinner														
Adjusted†,‡	−1·2	−2·9, 0·5	−0·7	−1·5, 0·2	−0·1	−0·5, 0·2	**−1·9**	**−3·4, −0·3**	−1·3	−3·4, 0·8	0·0	−0·1, 0·1	**−0·1**	**−0·1, 0·0**
Intra§	−1·2	−3·1, 0·7	−0·7	−1·7, 0·3	−0·2	−0·6, 0·3	−1·3	−3·1, 0·6	−1·2	−3·8, 1·5	0·0	−0·1, 0·1	0·0	−0·1, 0·1
Inter‖	−1·1	−4·7, 2·4	−0·7	−2·3, 1·0	−0·1	−0·7, 0·6	**−3·3**	**−6·0, −0·5**	−1·6	−5·0, 1·9	0·1	−0·1, 0·2	**−0·1**	**−0·2, −0·1**
Snacking														
Adjusted†,‡	1·3	−0·2, 2·8	0·7	−0·1, 1·4	0·0	−0·3, 0·3	**2·0**	**0·6, 3·3**	−0·5	−2·3, 1·4	0·0	−0·1, 0·0	**0·1**	**0·0, 0·1**
Intra§	1·4	−0·3, 3·1	0·8	−0·1, 1·7	−0·1	−0·4, 0·3	1·4	−0·2, 3·1	−1·8	−4·1, 0·6	−0·1	−0·2, 0·0	0·0	−0·1, 0·0
Inter‖	0·9	−1·9, 3·7	0·4	−1·0, 1·7	0·2	−0·3, 0·7	**3·1**	**0·9, 5·3**	1·4	−1·4, 4·2	0·0	−0·1, 0·1	**0·1**	**0·1, 0·2**

EN %, energy percentage; CIS, Checklist Individual Strength; EORTC QLQ-C30, European Organisation for the Research and Treatment of Cancer Quality of Life Questionnaire.

* The *β*-coefficients indicate the overall longitudinal difference in the outcome score using linear mixed models.

† Linear mixed models adjusted for sex (male/female), age at enrollment (years), time since end of treatment (weeks), chemotherapy (yes/no), comorbidities (0, 1, ≥2), BMI (kg/m^2^), diabetes (yes/no), moderate-to-vigorous physical activity (min/week), energy intake (kcal/week), stoma (yes/no), diet quality (WCRF-score), prolonged sedentary time (h/d) and alcohol intake (g/d). For the inflammatory summary *Z*-score (based on IL6, IL8, IL10, TNFα and hsCRP) associations were additionally adjusted for the use of non-steroidal anti-inflammatory drugs (yes/no).

‡ A random slope was added to the model for breakfast, lunch and dinner EN % with sleep duration; and lunch and snack EN % with the summary *Z*-score for inflammatory markers; no random slope was added to the models for all other associations (see Methods section).

§ The *β*-coefficients indicate the change in the outcome score over time (when exposure increases with 1-point between time-points from 6 weeks to 24 months post-treatment) within individuals.

‖ The *β*-coefficients indicate the difference in the outcome score between individuals across all time-points from 6 weeks to 24 months post-treatment.

Values in bold are statistically significant (*P* < 0·05).

Separate models testing inter- and intra-individual associations illustrated that the associations for the percentage of daily energy consumed during dinner and snacking between meals with fatigue (EORTC QLQ-C30) and inflammatory markers were mainly driven by the inter-individual component. This indicates that a higher average percentage of energy intake during dinner between individuals, and not changes in the percentage within individuals over time, was predominantly associated with less fatigue (*β*: −3·3; 95 % CI (−6·0, −0·5)) and less inflammation (*β*: −0·1; 95 % CI (−0·2, −0·1)) over time (Table 3). Similarly, the observed significant associations between the percentage of daily energy consumed during snacking between meals and fatigue (EORTC QLQ-C30) and inflammatory markers were mainly driven by the inter-individual component (*β*: 3·1; 95 % CI (0·9, 5·3)); and *β*: 0·1; 95 % CI (0·1, 0·2)), respectively). The observed association between the percentage of daily energy consumed during breakfast and sleep duration was mainly driven by the intra-individual component (*β*: 0·2; 95 % CI (0·0, 0·3)).

#### Irregular meal energy intake and irregular meal clock times

No consistent significant longitudinal associations were observed between irregularity scores based on meal energy intake and sleep quality, fatigue and inflammatory markers from 6 weeks to 24 months post-treatment (online Supplementary Table 1). Although mostly non-significant, a higher irregularity score based on the energy intake of breakfast, lunch and dinner resulted on average in slightly higher insomnia scores and fatigue scores on both scales. Only the associations between higher irregularity scores based on the energy intake of dinner and both higher fatigue (only EORTC QLQ-C30 subscale) and insomnia were statistically significant. A significant longitudinal association was observed between a higher irregularity of clock time of breakfast with a lower sleep duration (*β* per each hour difference from average clock time breakfast: −0·3; 95 % CI (−0·6, −0·1)). This association was predominantly driven by the intra-individual component, indicating that an increasing irregularity score within individuals over time was associated with a shorter sleep duration (*β*: −0·5; 95 % CI (−0·7, −0·3)). Other changes in irregularity scores based on clock times were not longitudinally associated with sleep quality, fatigue (all subscales) or inflammatory markers (online Supplementary Table 2).

#### Time window of energetic intake and relative meal frequency

For the TW of energetic intake, the confounder-adjusted longitudinal associations from 6 weeks to 24 months post-treatment showed that a longer TW was significantly associated with decreases in fatigue (both EORTC QLQ-C30 and CIS) and insomnia, a shorter sleep duration and lower levels of inflammatory markers (Table 4). For example, a longer TW of energetic intake was associated with decreased total fatigue (*β* per hour increase in TW: −2·8; 95 % CI (−4·2, −1·4)), subjective fatigue (*β*: −1·1; 95 % CI (−1·8, −0·4) and activity-related fatigue (*β*: −0·7; 95 % CI (−1·0, −0·4)), as measured by the CIS. A longer TW was also associated with a lower EORTC-fatigue (*β*: −1·6; 95 % CI (−3·0, −0·3)) and insomnia (*β*: −3·7; 95 % CI (−5·5, −2·0)). Most of the described associations were mainly driven by the inter-individual component. For activity-related fatigue (CIS) and sleep duration, the intra-individual association was also significant (Table 4). No significant overall longitudinal associations were found between the irregularity of the TW of energetic intake and outcomes (Table 4).

**Table 4.** Longitudinal associations of time window of energetic intake and meal frequency with sleep quality, fatigue and inflammatory markers between 6 weeks and 24 months post-treatment

Time window (TW) of energetic intake	CIS						EORTC-QLQ-C30				Dietary record		Inflammatory markers	
	Total fatigue (20–140)		Subjective fatigue (8–56)		Activity fatigue (3–21)		Fatigue (0–100)		Insomnia (0–100)		Sleep duration		Summary *Z*-score	
	*β* *	95 % CI	*β*	95 % CI	*β*	95 % CI	*β*	95 % CI	*β*	95 % CI	*β*	95 % CI	*β*	95 % CI
For each hour increase														
Adjusted†,‡	**−2·8**	**−4·2, −1·4**	**−1·1**	**−1·8, −0·4**	**−0·7**	**−1·0, −0·4**	**−1·6**	**−3·0, −0·3**	**−3·7**	**−5·5, −2·0**	**−0·5**	**−0·6, −0·4**	**−0·1**	**−0·1, 0·0**
Intra§	−1·6	−3·3, 0·0	−0·5	−1·3, 0·3	**−0·5**	**−0·9, −0·2**	−0·5	−2·0, 1·0	**−3·0**	**−5·2, −0·8**	**−0·4**	**−0·5, −0·3**	**−0·1**	**−0·1, 0·0**
Inter‖	**−6·1**	**−8·8, −3·3**	**−2·6**	**−3·8, −1·3**	**−1·1**	**−1·6, −0·6**	**−3·5**	**−5·6, −1·3**	**−4·8**	**−7·4, −2·1**	**−0·6**	**−0·7, −0·5**	0·0	−0·1, 0·0
Irregularity TW across days: for each hour difference from average time window of energetic intake														
Adjusted†,‡	−1·6	−4·2, 1·0	−0·7	−2·0, 0·6	−0·1	−0·7, 0·4	0·1	−2·2, 2·4	−1·3	−4·6, 2·0	−0·1	−0·3, 0·0	0·0	−0·1, 0·1
Intra§	**−2·9**	**−5·7, −0·1**	−1·3	−2·7, 0·1	−0·4	−1·0, 0·2	−0·3	−2·8, 2·3	−1·4	−5·2, 2·4	**−0·2**	**−0·3, −0·1**	0·0	−0·1, 0·1
Inter‖	5·7	−0·9, 12·4	2·1	−1·0, 5·2	1·1	−0·1, 2·4	1·5	−3·7, 6·7	−1·1	−7·6, 5·5	0·1	−0·2, 0·4	0·0	−0·1, 0·2
Meal frequency: for each 10 % increase of maximum total eating moments reported														
Adjusted†,‡	**−1·8**	**−3·5, −0·2**	−0·4	−1·2, 0·4	**−0·4**	**−0·7, −0·1**	**−1·8**	**−3·5, −0·1**	**−2·0**	**−4·0, 0·0**	**−0·1**	**−0·2, 0·0**	0·0	−0·1, 0·0
Intra§	−1·0	−3·0, 0·9	−0·1	−1·1, 0·9	−0·3	−0·7, 0·2	−1·5	−3·3, 0·3	−1·4	−4·1, 1·3	**−0·1**	**−0·2, 0·0**	0·0	−0·1, 0·1
Inter‖	**−3·7**	**−6·6, −0·8**	−1·1	−2·5, 0·3	**−0·6**	**−1·2, −0·1**	−1·5	−3·8, 0·9	−2·7	−5·6, 0·2	−0·1	−0·2, 0·0	0·0	−0·1, 0·0

TW, time window; CIS, Checklist Individual Strength; EORTC QLQ-C30, European Organisation for the Research and Treatment of Cancer Quality of Life Questionnaire.

* The *β*-coefficients indicate the overall longitudinal difference in the outcome score using linear mixed models.

† Linear mixed models adjusted for sex (male/female), age at enrollment (years), time since end of treatment (weeks), chemotherapy (yes/no), comorbidities (0, 1, ≥2), BMI (kg/m^2^), diabetes (yes/no), moderate-to-vigorous physical activity (min/week), energy intake (kcal/week), stoma (yes/no), diet quality (WCRF-score), prolonged sedentary time (h/d) and alcohol intake (g/d). For the inflammatory summary *Z*-score (based on IL6, IL8, IL10, TNFα and hsCRP), associations were additionally adjusted for the use of non-steroidal anti-inflammatory drugs (yes/no).

‡ A random slope was added to the model for the time window of energetic intake with fatigue (EORTC) and sleep duration; irregularity of the time window of energetic intake with sleep duration and for meal frequency with fatigue (EORTC) and the summary *Z*-score for inflammatory markers; no random slope was added to the models for all other associations (see Methods section).

§ The *β*-coefficients indicate the change in the outcome score over time (when exposure increases with 1-point between time-points from 6 weeks to 24 months post-treatment) within individuals.

‖ The *β*-coefficients indicate the difference in the outcome score between individuals across all time points from 6 weeks to 24 months post-treatment.

Values in bold are statistically significant (*P* < 0·05).

Table 4 shows that a higher relative meal frequency (out of a maximum of six meal slots/d that could be reported) was significantly associated with lower fatigue over time on both the CIS (*β* per 10 % of total meals slots reported: −1·8; 95 % CI (−3·5, −0·2)) and the EORTC-scale (*β*: −1·8; (−3·5, −0·1)). Additionally, a higher relative meal frequency was significantly associated with a lower insomnia score (*β*: −2·0; 95 % CI (−4·0, 0·0)) and a shorter sleep duration (*β*: −0·1; 95 % CI (−0·2, 0·0)). Except for the outcome sleep duration, these overall associations were predominantly driven by the inter-individual component.

### Sensitivity and time-lag analysis

In the time-lag analysis, in which exposures were coupled with outcomes at subsequent post-treatment time points, approximately equal overall and disaggregated longitudinal associations were observed (data not shown). In the sensitivity analysis, after omitting eating occasions with less than 50 kcal, we found similar associations with the outcomes in confounder-adjusted linear mixed models (data not shown).

## Discussion

In this prospective cohort of stage I-III CRC survivors, longitudinal analyses thoroughly adjusted for potential confounders showed that a longer TW of energetic intake was associated with decreases in fatigue, sleep duration, insomnia and inflammatory markers between 6 weeks and 24 months post-treatment. Similarly, a higher relative meal frequency was associated with decreases in insomnia and sleep duration and a decrease in fatigue over time (except for the subscale subjective fatigue where the decrease was non-significant). Associations for the TW of energetic intake and relative meal frequency appeared to be mainly driven by between-person differences over time and not by increases within individuals over time. This indicates that individuals with on average a longer TW of energetic intake or higher relative meal frequency over time predominantly had better outcomes than individuals with a shorter TW and lower relative meal frequency. No meaningful patterns in significant associations were observed for irregularity scores based on meal energy intake, meal clock time and the TW of energetic intake.

To our knowledge, this is the first longitudinal study looking into when, how often and how regular CRC patients consume food across the day and on repeated days and how this is related to patient-reported outcomes related to circadian rhythms such as fatigue and sleep. Two previous studies have investigated the effect of a shortened TW of energetic intake, or time-restricted eating, in breast cancer survivors. More specifically, O’Donnell *et al*.^(46)^ observed that when female breast cancer survivors with a median time of 4·5 years post-diagnosis adhered to a TW of energetic intake of ≤11 h (13-h overnight fasting), improvements occurred in anxiety after 6 weeks and in fatigue, anxiety, depression and BMI after 12 weeks^(46)^. In addition, Kleckner *et al*.^(47)^ found that cancer-related fatigue among mainly breast cancer survivors 4–60 months post-cancer treatment decreased after 2 weeks following a self-selected 10-h TW of beverage and food intake^(47)^. Our results appear not to be in line with these observations in breast cancer survivors. We did not observe any significant associations between intra-individual changes over time in TW of energetic intake and fatigue. Furthermore, participants having a longer TW of energetic intake had on average a larger decrease in fatigue compared to participants with a shorter TW. The lack of a control group in abovementioned studies in breast cancer patients could potentially explain the observed differences in findings, as we observed that fatigue decreased on average over time in our entire population. Additionally, the average TW of energetic intake of our study population was approximately 11·5 h, which in turn almost equals the 13-h overnight fast as described by O’Donnell *et al*.^(46)^ Given this relatively long average overnight fast observed in our population, it would be interesting to investigate if similar results would have been found if the average overnight fast was shorter, and consequently the average TW of energetic intake longer.

The majority of research focusing on the TW of energetic intake primarily looked into effects on body composition, obesity and metabolism^(48)^. In general, these studies found that a shorter TW of energetic intake, and therefore a longer overnight fast, was associated with improved glucose control in participants with and without type 2 diabetes (T2D)^(49,50)^. Additionally, there is preliminary evidence suggesting that a 10-h TW of energetic intake may be a feasible strategy to improve insulin sensitivity and to reduce energy intake in T2D patients^(51)^. The described underlying mechanism here is that longer fasting periods promote regulation of the central circadian clock in the superchiasmatic nucleus and peripheral clocks in metabolically active tissues^(48,52)^. This may result in less metabolic stress within cells, and therefore less inflammation^(48,53)^. In our study, we observed that a longer TW of energetic intake was longitudinally associated with significantly decreased fatigue, insomnia and inflammation. It is difficult to compare our results to the above-mentioned studies, because our population of CRC survivors is considerably different, consisting for a large part of elderly (>70 years) and frail individuals with comorbidities. In addition, we looked at patient-reported outcomes that are partly subjective. Consequently, it is possible that in our population of CRC survivors other mechanisms or factors play a role. For example, a longer TW of energetic intake might be a proxy for better overall well-being which is associated with less fatigue and better sleep quality, instead of directly causing the observed reductions in fatigue and sleep quality. This might also be supported by the fact that the inverse association between the TW of energetic intake and fatigue and sleep quality was mainly driven by inter-individual differences rather than intra-individual changes over time. Nevertheless, time-lag analyses provided similar results, providing some additional support for our finding that a longer TW of energetic intake may favorably be associated with fatigue, sleep quality and inflammation among CRC survivors.

As peripheral clock systems can be influenced by food consumption^(14)^, a well time-timed dietary intake could align peripheral clocks to the central circadian clock, potentially improving adverse health outcomes associated with a misalignment between clocks^(22,23)^. In this regard, previous studies in healthy populations have found that higher irregularity of energy intake during meals was associated with increased cardiovascular risk factors and the risk for metabolic syndrome^(26,54–56)^. In addition, a review focusing on the influence of meal frequency and timing on health highlighted that consuming larger proportions of energy earlier in the day and a reduced meal frequency of three meals/d may provide physiological benefits, including among others an improved circadian rhythm and reduced inflammation^(27)^. In our population of CRC survivors, we did not find significant longitudinal associations of irregularity scores based on energy intake and clock times of meals with sleep quality, fatigue and inflammatory markers. For meal energy contribution, we showed that a higher proportion of energy consumed during dinner was associated with lower fatigue, whereas a higher proportion of energy consumed outside of the main meals (snacking) was associated with higher fatigue. However, these results should be interpreted with caution, as associations with fatigue were only statistically significant when measured by the EORTC-QLQ-C30 scale. Finally, concerning meal frequency, our results showed that CRC survivors with a higher relative meal frequency (out of a maximum of six meals that could be reported) reported significant lower fatigue (except for the CIS subscale subjective fatigue) and a higher sleep quality. Of note, participants in our study could only report their total food and beverage intake across a maximum of six meals slots/d in 7-d food diaries, while in reality their true meal frequency was likely higher with on average six to seven daily intake occasions being reported in the general population of the Netherlands^(57)^. In addition, some participants may have had longer periods of continuous consumption of food (grazing) in between meals. This is justified by the observation that approximately 75 percent of participants reported consumption of calories during all available six meal slots in at least half of their available dietary record days, suggesting a potential ceiling effect. Consequently, there remains some uncertainty whether similar associations would have been observed if participants were given the opportunity to report the actual daily number of eating occasions instead of being restricted by a maximum of six.

This study features several strengths and limitations. The first strength is that we used a large prospective cohort of CRC survivors with repeated measurements, allowing analysis of changes over a relatively long follow-up time. Second, a major strength of the current study is the availability of 7-d dietary records, providing extensive quantitative data on food intake and clock times of major meals, enabling the detailed operationalisation of several chrono-nutrition variables. In general, dietary records are more accurate and less prone to recall bias than FFQ data commonly used in large observational studies^(58)^. In addition, the assessment of chrono-nutrition across multiple days resulted in a higher reliability of the dietary data^(58)^. Third, high response rates (>90 %) were observed for all post-treatment time points, resulting in low amounts of missing data. Fourth, extensive data on a wide range of potential confounders were available and accounted for in the analyses. Finally, the longitudinal nature of the current study allowed for the use of mixed models, making it possible to disaggregate the overall association into inter- and intra-individual associations, providing valuable additional insight into the nature of longitudinal relations. However, the study also has certain limitations that should be considered. Due to the observational nature of our study, we cannot be sure about the directionality of observed associations between chrono-nutrition variables and sleep quality, fatigue and inflammatory markers. Intervention and other observational studies with larger contrasts in chrono-nutrition variables are needed to be sure about the direction of associations and to infer causality. Moreover, no clock times were reported for meal slots outside of the main meals including breakfast, lunch and dinner. Therefore, in case of reported consumption after dinner, the clock time of last energetic intake had to be estimated to be able to calculate the TW. This could potentially have resulted in a under- or overestimation of the TW of energetic intake, which may have influenced the observed results. Therefore, it is important that future studies are conducted to validate our findings. Furthermore, the limited response rate at baseline (45 %) and potential non-random loss to follow-up might have resulted in selection bias. We observed that participants with a low education seemed to be slightly less likely to remain in the study compared to participants with a high education (Table 1). Additionally, although there was high agreement between the triaxial MOX activity meter and reported wake and bedtimes, we did not account for sleep onset latency. This implies that there could be differences between reported time of going to bed and the time participants actually fall asleep. Consequently, sleep duration may be slightly overestimated in the current study. In addition, no information on the chronotype of participants was collected during this study. Therefore, we were not able to control for inter-individual differences in circadian rhythms. Ideally, future studies should measure participants’ chronotype to be able to accurately investigate if nutritional intake of individuals is in accordance to their circadian rhythm on an individual level. Finally, the possibility of residual confounding and chance findings due to the high number of analyses cannot be ruled out.

In conclusion, the findings of this study underline that aspects related to the timing of food intake (chrono-nutrition) are of potential relevance in CRC survivors. Our main finding is that in our population, a longer TW of energetic intake was observed to be longitudinally associated with reduced fatigue and inflammation and with improved sleep quality in the first two years after the end of CRC treatment. Future studies are needed to confirm findings of the current study and to further investigate the relation between chrono-nutrition variables and patient reported and other health outcomes in CRC survivors. This knowledge could ultimately result in updating individual evidence-based dietary recommendations to improve CRC survivors’ quality of life by incorporating time-related aspects of diet next to the quality of the diet.

## Supporting information

Chong et al. supplementary material Chong et al. supplementary material

## Supplementary material

For supplementary material/s referred to in this article, please visit https://doi.org/10.1017/S0007114523002714
