# Supplementary material for: Longitudinal associations of circadian eating patterns with sleep quality, fatigue and inflammation in colorectal cancer survivors up to 24 months post-treatment
Source: Br J Nutr. 2023 Nov 28;131(7):1166–80. doi: 10.1017/S0007114523002714 (PMC10918519; doi:10.1017/S0007114523002714)
Supplement: Chong et al. supplementary material [file S0007114523002714sup001.zip › SUPP~U$T.PDF]

Supplementary Table 1. Longitudinal associations of meal irregularity score based on variability in energy intake across days with sleep quality, fatigue, and inflammatory markers between 6 weeks and 24 months post-treatment.

**Irregular Meal Energy Intake**	**CIS**			**EORTC-QLQ-C30**		**Dietary record**	**Inflammatory markers**
For each average 10EN% change across days	Total fatigue (20 – 140)	Subjective Fatigue (8 – 56)	Activity Fatigue (3 – 21)	Fatigue (0 -100)	Insomnia (0-100)	Sleep duration	Summary z-score
	β[1] (95% CI)	β (95% CI)	β (95% CI)	β (95% CI)	β (95% CI)	β (95% CI)	β (95% CI)
**Breakfast**							
Adjusted[2]	0.1 (-0.6 – 0.7)	0.0 (-0.3 – 0.3)	0.1 (0.0 – 0.3)	0.3 (-0.3 – 0.9)	0.1 (-0.8 – 0.9)	0.0 (0.0 – 0.1)	0.0 (0.0 – 0.0)
Intra[4]	-0.1 (-0.8 – 0.6)	0.0 (-0.4 – 0.3)	0.1 (0.0 – 0.3)	0.2 (-0.5 – 0.8)	-0.4 (-1.4 – 0.6)	0.0 (0.0 – 0.1)	0.0 (-0.1 – 0.0)
Inter[5]	0.7 (-0.7 – 2.2)	0.2 (-0.5 – 0.9)	0.1 (-0.2 – 0.4)	0.6 (-0.6 – 1.7)	1.0 (-0.4 – 2.4)	0.1 (0.0 – 0.1)	0.0 (0.0 – 0.1)
**Lunch**							
Adjusted[2,3]	0.3 (-0.1 – 0.8)	0.1 (-0.1 – 0.3)	0.0 (-0.1 – 0.1)	0.3 (-0.1 – 0.7)	0.2 (-0.3 – 0.8)	0.0 (0.0 – 0.0)	0.0 (0.0 – 0.0)
Intra[4]	0.2 (-0.3 – 0.7)	0.0 (-0.2 – 0.2)	0.0 (-0.1 – 0.1)	0.2 (-0.3 – 0.6)	-0.2 (-0.9 – 0.5)	0.0 (0.0 – 0.0)	0.0 (0.0 – 0.0)
Inter[5]	**1.0 (0.0 – 2.1)**	0.4 (-0.1 – 0.9)	0.2 (0.0 – 0.4)	0.7 (-0.1 – 1.5)	**1.2 (0.2 – 2.2)**	**0.1 (0.0 – 0.1)**	**0.0 (0.0 – 0.1)**
**Dinner**							
Adjusted[2,3]	0.6 (-0.2 – 1.5)	0.4 (0.0 – 0.8)	0.2 (0.0 – 0.3)	**0.9 (0.1 – 1.7)**	**1.8 (0.6 – 3.1)**	0.0 (0.0 – 0.0)	0.0 (0.0 – 0.1)
Intra[4]	0.5 (-0.4 – 1.5)	0.4 (-0.1 – 0.8)	0.1 (-0.1 – 0.3)	0.8 (0.0 – 1.7)	1.1 (-0.1 – 2.3)	0.0 (0.0 – 0.1)	0.0 (0.0 – 0.1)
Inter[5]	1.2 (-0.9 – 3.4)	0.3 (-0.7 – 1.4)	0.3 (-0.1 – 0.7)	0.7 (-1.0 – 2.4)	**2.1 (0.0 – 4.2)**	0.0 (-0.1 – 0.1)	**0.1 (0.0 – 0.1)**
**Snacking**							
Adjusted[2,3]	0.2 (-0.3 – 0.6)	0.0 (-0.3 – 0.2)	0.0 (-0.1 – 0.1)	-0.2 (-0.7 – 0.2)	0.6 (-0.1 – 1.2)	0.0 (0.0 – 0.0)	0.0 (0.0 – 0.0)
Intra[4]	0.1 (-0.4 – 0.7)	0.0 (-0.3 – 0.2)	0.1 (-0.1 – 0.2)	-0.1 (-0.6 – 0.3)	0.6 (-0.1 – 1.3)	0.0 (0.0 – 0.0)	0.0 (0.0 – 0.0)
Inter[5]	0.2 (-1.0 – 1.5)	0.0 (-0.6 – 0.6)	0.0 (-0.3 – 0.2)	-0.5 (-1.5 – 0.5)	0.4 (-0.9 – 1.7)	0.0 (0.0 – 0.1)	**0.0 (-0.1 – 0.0)**

Irregular Meal Energy Intake	CIS			EORTC-QLQ-C30		Dietary record	Inflammatory markers
For each average 10EN% change across days	Total fatigue (20 – 140)	Subjective Fatigue (8 – 56)	Activity Fatigue (3 – 21)	Fatigue (0 -100)	Insomnia (0-100)	Sleep duration	Summary z-score
	β[1] (95% CI)	β (95% CI)	β (95% CI)	β (95% CI)	β (95% CI)	β (95% CI)	β (95% CI)
**Entire day**							
Adjusted[2]	0.2 (-1.5 – 1.8)	0.0 (-0.8 – 0.8)	0.2 (-0.1 – 0.6)	-0.4 (-1.9 – 1.1)	1.4 (-0.8 – 3.6)	0.1 (0.0 – 0.1)	0.0 (-0.1 – 0.0)
Intra[4]	0.2 (-1.6 – 1.9)	0.0 (-0.9 – 0.9)	0.2 (-0.2 – 0.6)	-0.6 (-2.2 – 1.0)	1.1 (-1.3 – 3.4)	0.1 (0.0 – 0.1)	0.0 (-0.1 – 0.1)
Inter[5]	0.0 (-5.1 - 5.0)	0.2 (-2.2 – 2.6)	0.3 (-0.7 – 1.3)	0.9 (-3.2 – 4.9)	3.1 (-2.0 – 8.1)	0.1 (-0.1 – 0.3)	0.0 (-0.1 – 0.1)

Abbreviations: EN%, energy percentage; CIS, Checklist Individual Strength; EORTC QLQ-C30, European Organization for the Research and Treatment of Cancer Quality of Life Questionnaire.

[1] *The β-coefficients indicate the overall longitudinal difference in the outcome score using linear mixed models.*

[2] *Linear mixed-models adjusted for sex (male/female), age at enrollment (years), weeks since end of treatment (weeks), chemotherapy (yes/no), comorbidities (0, 1, ≥2), BMI (kg/m²), diabetes (yes/no), moderate-to-vigorous physical activity (min/week), energy intake (kcal/week), stoma (yes/no), diet quality (WCRF-score), prolonged sedentary time (hours/day), and alcohol intake (g/day). For the inflammatory summary z-score (based on IL-6, IL-8, IL-10, TNFa, and hsCRP), associations were additionally adjusted for the use of non-steroidal anti-inflammatory drugs (yes/no).*

[3]*A random slope was added to the model for irregular lunch energy intake score with sleep duration and the summary z-score for inflammatory markers; irregular dinner energy intake score with fatigue (EORTC) and insomnia (EORTC); and for irregular snacking energy intake score with sleep duration and the summary z-score for inflammatory markers; no random slope was added to the models for all other associations (see Methods section).*

[4] *The β-coefficients indicate the change in the outcome score over time (when exposure increases with 1-point between time-points from 6 weeks to 24 months post-treatment) within individuals.*

[5] *The β-coefficients indicate the difference in the outcome score between individuals across all time-points from 6 weeks to 24 months post-treatment.*

Supplementary Table 2. Longitudinal associations of meal irregularity score based on variability in clock-times of meal intake across days with sleep quality, fatigue, and inflammatory markers between 6 weeks and 24 months post-treatment.

**Irregular Clock-Time Meal**	**CIS**			**EORTC-QLQ-C30**		**Dietary record**	**Inflammatory markers**
For each hour difference from average clock-time	Total fatigue (20 – 140) β^1 (95% CI)	Subjective Fatigue (8 – 56) β (95% CI)	Activity Fatigue (3 – 21) β (95% CI)	Fatigue (0 -100) β (95% CI)	Insomnia (0-100) β (95% CI)	Sleep duration β (95% CI)	Summary z-score β (95% CI)
**Breakfast**							
Adjusted[2,3]	-0.8 (-4.5 – 2.8)	0.1 (-1.7 – 1.9)	-0.3 (-1.1 – 0.4)	-0.7 (-3.9 – 2.6)	-3.5 (-8.1 – 1.1)	**-0.3 (-0.6 – -0.1)**	-0.1 (-0.2 – 0.1)
Intra[4]	-1.8 (-5.9 – 2.2)	-0.6 (-2.6 – 1.4)	-0.5 (-1.4 – 0.4)	-2.1 (-5.8 – 1.6)	-5.0 (-10.4 – 0.5)	**-0.5 (-0.7 – -0.3)**	**-0.2 (-0.3 – 0.0)**
Inter[5]	3.6 (-4.7 – 11.9)	2.8 (-1.1 – 6.7)	0.2 (-1.4 – 1.8)	3.9 (-2.6 – 10.4)	-0.1 (-8.3 – 8.0)	0.0 (-0.4 – 0.4)	0.1 (-0.1 – 0.2)
**Lunch**							
Adjusted[2]	2.7 (-1.5 – 6.9)	1.2 (-0.9 – 3.3)	0.6 (-0.3 – 1.5)	0.2 (-3.5 – 3.9)	-0.5 (-5.8 – 4.8)	0.0 (-0.2 – 0.2)	0.0 (-0.1 – 0.2)
Intra[4]	1.7 (-2.9 – 6.3)	0.8 (-1.5 – 3.1)	0.3 (-0.7 – 1.3)	-1.2 (-5.4 – 2.9)	-1.0 (-7.2 – 5.2)	0.0 (-0.3 – 0.2)	0.0 (-0.2 – 0.1)
Inter[5]	7.6 (-3.0 – 18.2)	3.0 (-2.0 – 8.1)	1.7 (-0.3 – 3.8)	6.2 (-2.4 – 14.8)	1.0 (-9.6 – 11.6)	0.1 (-0.4 – 0.5)	0.1 (-0.1 – 0.4)
**Dinner**							
Adjusted[2]	-1.0 (-4.3 – 2.3)	-0.3 (-1.9 – 1.3)	-0.1 (-0.8 – 0.7)	1.8 (-1.2 – 4.7)	1.0 (-3.3 – 5.3)	0.0 (-0.1 – 0.2)	-0.1 (-0.2 – 0.0)
Intra[4]	-2.2 (-5.7 – 1.3)	-1.0 (-2.7 – 0.8)	-0.3 (-1.1 – 0.5)	0.2 (-3.0 – 3.4)	-1.2 (-6.0 – 3.6)	0.0 (-0.2 – 0.2)	**-0.1 (-0.3 – 0.0)**
Inter[5]	9.0 (-1.1 – 19.1)	4.7 (0.0 – 9.5)	1.5 (-0.5 – 3.4)	**11.6 (3.6 – 19.6)**	**10.6 (0.7 – 20.6)**	0.2 (-0.3 – 0.6)	0.1 (-0.1 – 0.4)

Abbreviations: CIS, Checklist Individual Strength; EORTC QLQ-C30, European Organization for the Research and Treatment of Cancer Quality of Life Questionnaire.

[1] The β-coefficients indicate the overall longitudinal difference in the outcome score using linear mixed models.

[2] Linear mixed-models adjusted for sex (male/female), age at enrollment (years), weeks since end of treatment (weeks), chemotherapy (yes/no), comorbidities (0, 1, ≥2), BMI (kg/m²), diabetes (yes/no), moderate-to-vigorous physical activity (min/week), energy intake (kcal/week), stoma (yes/no), diet quality (WCRF-score), prolonged sedentary time (hours/day), and alcohol intake (g/day). For the inflammatory summary z-score (based on IL-6, IL-8, IL-10, TNFa, and hsCRP), associations were additionally adjusted for the use of non-steroidal anti-inflammatory drugs (yes/no).

[3] *A random slope was added to the model for irregular breakfast clock-time score with sleep duration no random slope was added to the models for all other associations (see Methods section).*
[4] *The β-coefficients indicate the change in the outcome score over time (when exposure increases with 1-point between time-points from 6 weeks to 24 months post-treatment) within individuals.*
[5] *The β-coefficients indicate the difference in the outcome score between individuals across all time-points from 6 weeks to 24 months post-treatment.*
